# Correlation of Clinical Course with Computed Tomography Findings and Biochemical Parameters at the Time of Admission in COVID-19 Patients

**DOI:** 10.5152/TJAR.2021.21175

**Published:** 2022-08-01

**Authors:** Ezel Yaltırık Bilgin, Erkan Bilgin, Hatice Fidan, Yıldıray Çelenk, Tuğba Tok

**Affiliations:** 1 Department of Radiology, Karadeniz Ereğli State Hospital, Zonguldak, Turkey; 2 Department of Anaesthesia and Intensive Care, Karadeniz Ereğli State Hospital, Zonguldak, Turkey; 3 Department of Emergency Medicine, Karadeniz Ereğli State Hospital, Zonguldak, Turkey; 4 Department of Infectious Disease, Karadeniz Ereğli State Hospital, Zonguldak, Turkey

**Keywords:** Computed tomography, COVID-19, inflammatory markers, intensive care, thorax

## Abstract

**Objective:**

The primary objective of our study was to evaluate the predictive performance of serum inflammatory markers and the semi-quantitative computed tomography severity scoring system on diagnosing the Covid 2019 disease and its course.

**Methods:**

Our study is a single-center retrospective cohort study. The data of 213 adults who were confirmed to have coronavirus disease 2019 by polymerase chain reaction tests in the period between April 2020 and August 2020 were evaluated. One hundred eighty four of these patients whose C-reactive protein, d-dimer, and ferritin levels, lymphocyte counts, and thoracic computed tomography images were obtained at the time of admission were included in the study. The semi-quantitative computed tomography severity score was calculated for all patients.

**Results:**

The median age of the 184 patients included in the study was 51.5 (19-91) years. The incidence of intensive care need and mortality was 10.3% (n = 19) and 5.4% (n = 10), respectively. The intensive care need and mortality rate was significantly correlated with higher thoracic computed tomography involvement scores at admission. There was a statistically significant and positive correlation between the computed tomography scores and the C-reactive protein, d-dimer, and ferritin levels. Older age (>65 years-old) and thoracic computed tomography scores of 11 and higher were independent factors correlated with need for intensive care.

**Conclusion:**

Serum inflammatory markers and semi-quantitative computed tomography severity scoring system were predictive in diagnosing the Covid 2019 disease and its course.

## Main Points

- The intensive care requirements and mortality were significantly higher in patients with higher thoracic CT scores at admission.
- The optimal positive cut-off values to determine the need for intensive care in coronavirus disease 2019 patients were 10.5 lung CT and age and 64 years, respectively.
- Serum C-reactive protein, d-dimer, and ferritin levels were statistically correlated, whereas serum lymphocyte levels were negatively correlated with the lung CT scores.

## Introduction

In December 2019, numerous cases of pneumonia, which were thought to have originated from a seafood market, were reported from Wuhan in China. The examinations of the samples collected from the respiratory tract confirmed that those pneumonia cases were caused by a novel coronavirus called novel coronavirus 2019 (2019-nCoV), coronavirus disease 2019 (COVID-19), or severe acute respiratory syndrome coronavirus-2 (SARS-CoV-2).^1^

Clinical manifestations of patients infected with SARS-CoV-2 range from mild nonspecific symptoms to severe pneumonia leading to organ damage and failure. Common symptoms are fever (77.4-98.6%), cough (59.4-81.8%), fatigue (38.1-69.6%), shortness of breath (3.2-55.0%), myalgia (11.1-34.8%), sputum production (28.2-56.5%), and headache (6.5-33.9%).^2^ In severe cases, organ dysfunction may occur including shock, acute respiratory distress syndrome, acute cardiac injury, acute kidney injury, and death.^1^ Furthermore, studies have shown that between 2% and 10% of cases have gastrointestinal complaints such as vomiting, diarrhea, and abdominal pain.^3^

Diagnosis of SARS-COV2 is based on clinical findings, molecular diagnosis of the viral genome by reverse transcriptase-polymerase chain reaction (RT-PCR), and findings obtained through chest radiography, CT, and serological blood tests. With the experience to date, the multiplex polymerase chain reaction test is accepted as the gold standard diagnostic method for SARS-CoV-2 but with low sensitivity ranging from 42% to 71%.^4^

The basic routine tests recommended for COVID-19 patients include complete blood count, tests investigating coagulation and fibrinolysis steps (prothrombin time, activated partial thromboplastin time, and d-dimers), and inflammation-related parameters (erythrocyte sedimentation rate [ESR], C-reactive protein [CRP], ferritin, and procalcitonin [PCT]).^5^ Inflammatory markers such as PCT, serum ferritin, ESR, CRP, and interleukin-6 (IL-6) have been reported to be significantly associated with high risks of developing severe SARS-COV2.^6^

CT findings were similar to those in viral pneumonia cases on radiological examination. Multifocal ground-glass opacities and peripheral areas of consolidation are the most common findings.^7^ Although the specificity of these CT findings for the diagnosis of SARS-COV2 is low, such findings are used as objective parameters in diagnosis and follow-up.

The course of the disease varies according to the patient. Clinicians should be aware of the potential for rapid deterioration in some patients, generally 1 week after the onset of the disease.

The primary objective of our study was to evaluate the performance of serum inflammatory markers and the semi-quantitative CT severity scoring system, which was developed to determine the severity of SARS-COV2, in making the diagnosis and predicting the burden and course of the disease.

## Methods

### Patient Population and Study Setup

Our study is a single-center retrospective cohort study. The local ethics committee approved this retrospective study (2020/22-22).

The data of 213 patients older than 18, who applied to our hospital due to a suspicion of SARS-COV2, and who were confirmed to have SARS-COV2 by PCR tests between April 2020 and August 2020 were evaluated. Of these patients, 184 patients whose CRP, D-dimer, ferritin levels, lymphocyte counts, and thoracic CT images were evaluated at the time of admission were included in the study. The patients were evaluated in 3 different age groups; 18-40, 40-65, and patients above 65 years of age.

### Clinical Information

The laboratory parameters tested at the time of admission, CRP, d-dimer, ferritin levels, and lymphocyte counts of the patients were recorded retrospectively. All patients were followed up under hospital conditions. The clinical course of the patients was evaluated in 3 separate groups. The patients discharged from the hospital, patients who needed intensive care, and patients who died.

### Computed Tomography Scanning and Image Analysis

All patients were evaluated using the same multidetector CT scanner (Somatom, Siemens, Germany). Computed tomography images of the patients were obtained, complying with appropriate infection prevention precautions. Computed tomography images were obtained with the patient in the supine position and at full inspiration. Standard thoracic CT presets of the device were used for scanning parameters. Images were reconstructed with a slice thickness of 5 mm. The reconstructed images were transferred to the workstation and a picture archiving and communication system. When needed, multiplanar reconstruction images were used, too.

Thoracic CT examinations of the patients were evaluated separately by 2 radiologists with more than 10 years of thoracic CT experience. In case of disagreement, a re-evaluation was made for the final decision.

**Figure 1.**
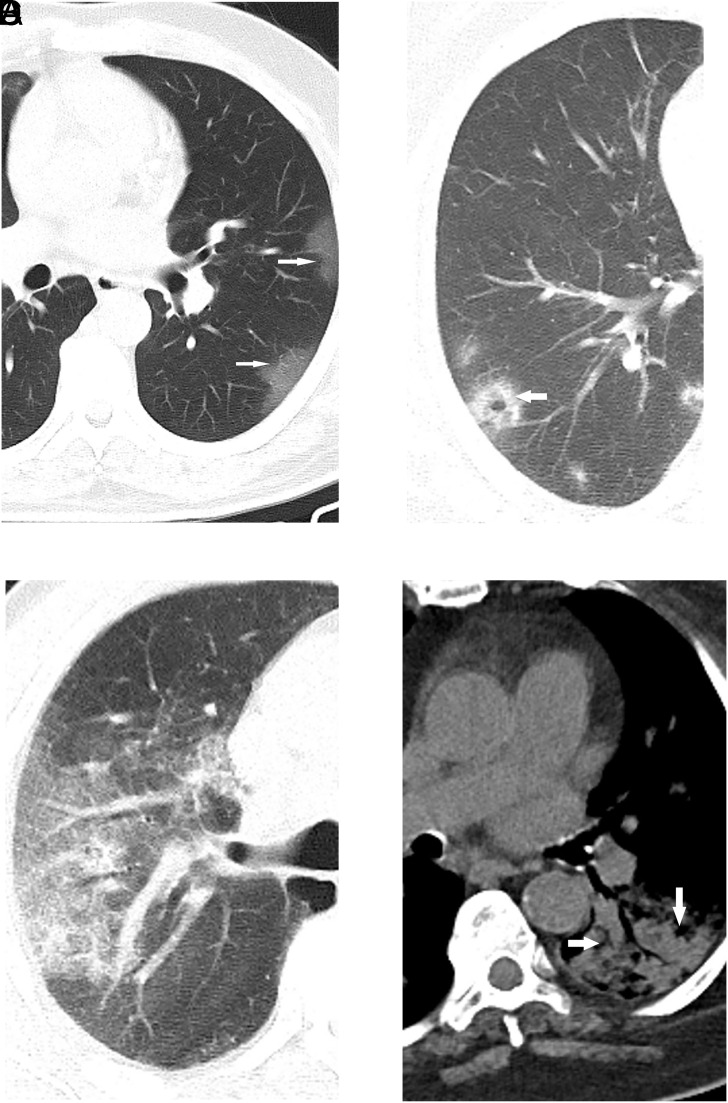
(A-D) Common CT involvement patterns in COVID-19 pneumonia. (A) Peripheral ground-glass opacity (arrows). (B) Reverse-halo sign is defined as a focal, rounded area of ground-glass attenuation surrounded by a ring of denser consolidation (arrow). (C) Crazy-paving refers to the appearance of ground-glass opacity with superimposed interlobular septal thickening and intralobular septal thickening. (D) Consolidation with air bronchograms (arrow). CT, computed tomography; COVID-19, coronavirus disease 2019.

**Figure 2. A:**
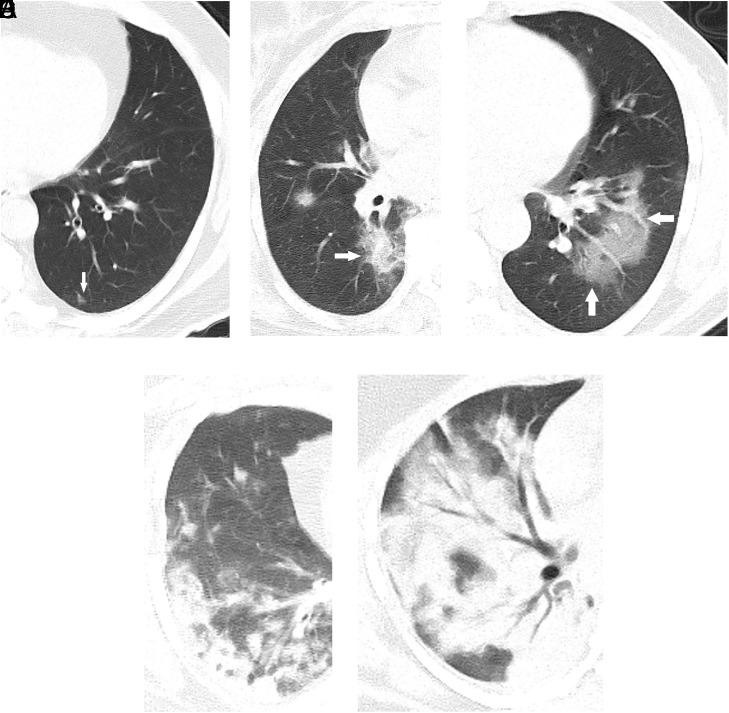
-E. CT involvement scores. (A) Score 1, (B) score 2, (C) score 3, (D) score 4, (E) score 5 involvement of related lobe. CT, computed tomography.

In light of the literature, the radiological pattern observed in the thoracic CT examination was classified as ground-glass opacity, crazy-paving pattern, and consolidation^8^ (Figure 1A-D). The semi-quantitative CT severity score suggested by Pan et al.^9^ was calculated for all patients with 5 lobes of the lung. The semi-quantitative CT severity scores are as follows: 0 = no involvement, 1 = less than 5% involvement, 2 = 5-25% involvement, 3 = 26-50% involvement, 4 = 51-75% involvement, and 5 = more than 75% involvement (Figure 2A-E). The CT severity scores ranged from 0 to 25 points, and were calculated by summing up the obtained score points attributed to each lung separately. Computed tomography severity scores of 0-8 were evaluated as mild involvement, 9-16 as moderate involvement, and 17-25 as severe involvement.

### Statistical Analysis

Statistical Package for the Social Sciences version 25.0 (IBM Corp.; Armonk, NY, USA) software was used for the statistical analysis. Besides descriptive statistical methods (number, percentage, mean, median, standard deviation, etc.), the Kruskal–Wallis test, Mann–Whitney *U* test, and Wilcoxon test were used to test the quantitative differences across the groups. In the Kruskal–Wallis test analysis, multiple comparisons of the groups with significant differences were performed using the Bonferroni correction. Chi-square (Pearson’s chi-square, continuity correction, and Fisher’s exact test) tests were used for the categorical data analysis across the groups. The level of correlation between the 2 variables was examined using Spearman’s correlation test. To predict the need for intensive care unit (ICU), receiver-operating characteristic (ROC) analysis was used to determine the optimal cut-off values for CT scores, age, CRP, d-dimer, and ferritin positivity. Logistic regression analysis was used to determine independent factors related to intensive care needs and mortality. The results were evaluated with a CI of 95% and at a significance level of *P* < .05.

## Results

Of the 184 patients included in the study, the median age was 51.5 (19-91) years, 28% (n = 52) were ≤40 years old, 50% (n = 92) were 41-64 years old, and 22% (n = 40) were ≥65 years old. Of the study patients, 52% (n = 95) were women. Intensive care need and mortality occurred in 10.3% (n = 19) and 5.4% (n = 10) of the patients, respectively.

On the thoracic CT images of the patients, a SARS-COV2 pneumonia image pattern at admission manifested ground-glass opacities in 120 patients (65%), crazy-paving in 15 patients (8%), consolidation findings in 8 patients (4%), and a normal view in 41 patients (22%). Computed tomography involvement scores were mild in 91 patients (49.5%), moderate in 49 patients (26.6%), and severe in 3 patients (1.6%). Selected biochemical parameters of the 184 patients included in the study were as follows: CRP, d-dimer, ferritin, and lymphocyte count mean numbers were 27.65, 707.64, 246.08, and 1.65, respectively.

According to the image patterns on thoracic CT images, there was a statistically significant difference in the age of 143 patients with abnormal findings as follows: median age was 53 (19-89) years in patients with a ground-glass view, 61 (37-88) years in patients with a crazy-paving view, and 67 (31-91) years in patients with a consolidation view (*P*  = .028). There were no statistically significant differences in gender distribution (*P* = .629). According to the abnormal image patterns on thoracic CT images, CRP, d-dimer, and ferritin levels were significantly different (CPR → K–W = 20.836, *P*   = <.001; D-dimer → K–W = 13.345, *P *  = .001; and ferritin → K–W = 6.133, *P*   = .047). Bonferroni corrections revealed that the differences occurred in CRP and d-dimer levels when the group with a ground-glass view was compared to the group with a crazy-paving view and the group with consolidation findings (*P * < .017). In the subgroup comparisons for ferritin values, a statistically significant difference was not found (*P* > .017). No statistically significant differences were found in lymphocyte counts according to the different thoracic CT image patterns (*P*   = .782). According to the thoracic CT image patterns, the need for intensive care (ground-glass: 10%, crazy-paving: 27%, and consolidation: 38%) and mortality rates (ground-glass: 5%, crazy-paving: 7%, and consolidation: 38%) were statistically significantly different ([for intensive care need, χ^2^ = 7.527; *P*   = .023] and [for mortality, χ^2^  = 12.183; *P*   = .002]) (Table 1).

Computed tomography involvement scores were lower in the 40 years age group compared to the other age groups (median CT score in the 40 years age group = 3 [1-11], in the 41-64 years group = 8 [1-21], and in the ≥ 65 years group = 10 [2-19]; *P*   = < .001). The correlation analysis revealed that CT scores increased significantly as age increased (*r * = 0.372; *P  * = < .001). The rate of patients with intensive care requirements and the rate of mortality had statistically significantly higher thoracic CT involvement scores at admission (median CT score of patients with ICR = 13 [7-21] vs 6 [1-16]; *P*   = < .001; median CT score of patients who died = 13 [11-18] vs 7 [1-21]; *P  * = .001). There was a statistically significant positive correlation (*r * = 0.24-0.67; *P* ≤ .001) between the CT involvement scores measured in all locations of both lobes of the lung and the CRP, d-dimer, and ferritin levels. A negative correlation (*r*  = 0.218; *P*  = .009) was found between lymphocyte counts and total thoracic CT involvement scores (Table 2).

Computed tomography involvement scores were statistically significantly different when the score of the superior right lobe of the lung was compared to the CT involvement scores of both the right and left inferior lobes ([R-superior vs R-inferior → *Z* = −5.415; *P* ≤ .001]; [R-superior vs L-inferior → *Z*  = −4.479; *P*  = .001]). There was a statistically significant difference, too, in the CT involvement scores of the superior left lobe and the inferior left lobe (L-superior vs L-inferior →*Z*  = −4.472; *P * = .001). Both the right and left involvement was more severe and inferiorly.

**Figure 3.**
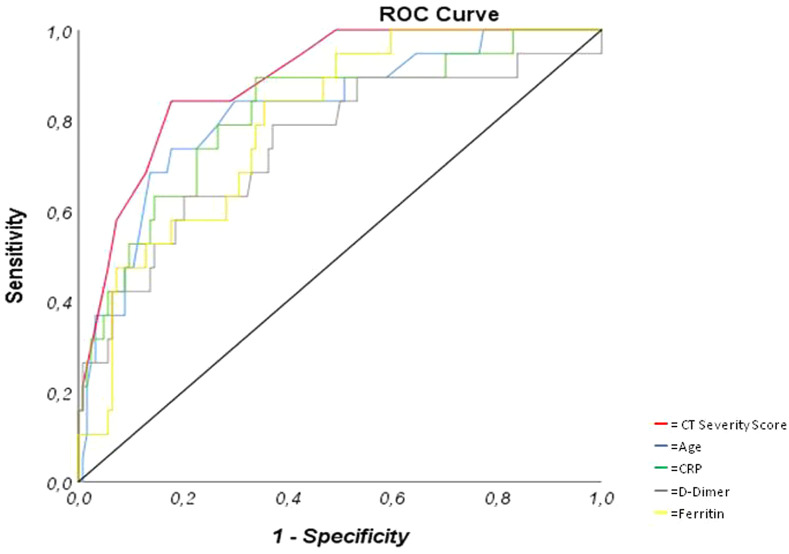
Positive cut-off limits of CT severity score, age, CRP, d-dimer, and ferritin values for determining the intensive care need in SARS-COV2 patients. CT, computed tomography; SARS-COV2, coronavirus disease 2019; CRP, C-reactive protein.

In the ROC analysis, the optimal positive cut-off values to determine the need for intensive care in COVID-19 patients were as follows: 10.5 for the lung CT involvement score (sensitivity: 0.842; specificity: 0.823; *P* ≤ .001); 64 years for age (sensitivity: 0.737; specificity: 0.823; *P*  = .001); 20.7 mg L^-1^ for CRP (sensitivity: 0.895; specificity: 0.661; *P*  = .001); 805 ng mL^-1^ for d-dimer (sensitivity: 0.632; specificity: 0.798; *P * = .001); and 211 ng mL^-1^ for ferritin (sensitivity: 0.842; specificity: 0.645; *P*  = .001) (Figure 3). The results of the multivariate logistic regression analysis revealed that the independently related factors with the need for intensive care were ages of 65 years old and older (OR = 5.80; *P * = .017) and thoracic CT involvement scores of 11 and higher (OR = 20.60; *P* ≤ .001). Independent factors associated with mortality were ages of 65 years old and over (OR = 7.88; *P*  = .034) and thoracic CT involvement scores (OR = 1.36; *P * = .012) (Table 3).

## Discussion

Severe acute respiratory syndrome coronavirus 2 is a betacoronavirus belonging to the Coronaviridae family and the order Nidovirales. Bats are thought to act as the reservoirs for SARS-CoV-2, an enveloped RNA virus.^10,11^

Patients can manifest varying disease courses ranging from an asymptomatic status or a mild course (up to 80%) to severe involvement with unilateral or bilateral pneumonia (approximately 15%) or a highly severe course with bilateral pneumonia and respiratory distress requiring ventilation support in the ICU (3%-5%).^12^

In some patients, clinical deterioration and intensive care needs may occur approximately 7-10 days after the onset of symptoms. The mortality rate in the general population ranges from 1.4% to 8%.^13^ In our study, the mortality rate was 5.4% and the need for intensive care was 10.3% in 184 patients with positive PCR tests. In 143 patients with positive PCR tests and CT findings, the mortality rate was 7% and the need for intensive care was 13.3%. In our district, where occupational pulmonary diseases due to mining and related industry are common, it receives the attention that mortality and the need for intensive care are within the normal ranges as reported in the literature despite being found close to the upper limits.

Despite several studies conducted to determine the parameters that predict a severe disease course and death, no strong evidence has been found yet.^14^

In a study on 171 patients, Zhou et al^14^ have shown that advanced age and d-dimer levels of more than 1 μg mL^-1^ at admission is associated with increased mortality. That study also reported that high levels of blood IL-6, cardiac troponin-I, lactate dehydrogenase, and lymphopenia were observed more frequently in severe SARS-COV2.

In a study conducted on 221 patients, Liu et al.^15^ observed that lymphopenia and low albumin levels were more common in patients older than 60 years old and that the rate of having a severe disease course was higher in this age group and longer recovery time was needed. Another finding of that study was the significantly longer mean disease duration in men than in women.

In a study on 168 patients manifesting a severe and critical disease course, Meng et al.^16^ found that the mortality rate was higher in men and patients older than 80. In that study, lymphopenia high lactate dehydrogenase levels, hemoglobin, hematocrit, ferritin, aspartate aminotransferase, alanine aminotransferase, CRP, and PCT were observed more frequently in male patients.

Computed tomography findings are observed to be similar to those of viral pneumonia. Multifocal ground-glass opacities and consolidation in the periphery are the most common findings.^17-18^ Studies in the literature have attempted to score CT findings objectively to evaluate the correlation of such scores with the clinical course.

The most common CT pattern in our study was the ground-glass opacity view, similar to the reports in the literature. On CT examinations, our patients’ most commonly involved lung lobes were the right and left lower lobes.

A study on 102 patients investigated the relationship between CT severity scores and the clinical course. The involvement was most common in the right and lower lobes. In that study, the 18 segments of both lungs were divided into 20 regions. Lung opacities in those all 20 lung regions were examined subjectively on CT images. Each zone was scored 0, 1, or 2 points depending on the degree of parenchymal opacification (0%, 1-50%, or 51-100%, respectively). The total CT severity was scored in values ranging from 0 to 40 points, which was the sum of the scores attributed to the 20 lung segment regions. In that study, CT severity scores were found high to be in severe diseases compared to diseases of mild and moderate severity. The cut-off score in severe cases was 19.5 points with 83.3% sensitivity and 94% specificity.^9^

In another study on 78 patients, investigators evaluated each of the 5 lobes of both lungs for the presence of inflammatory abnormalities. Each lobe was scored from 0 to 4 points depending on the percentage attributed to the respective lobe: 0 (0%), 1 (1-25%), 2 (26-50%), 3 (51-75%), or 4 (76-100%). Then the total severity score was calculated by summing up the scores of each of the 5 lobes. In that study, the cut-off score to predict a severe-critical disease course was 7.5, with a sensitivity of 82.6% and a specificity of 100%.

Our study found that serum CRP, d-dimer, and ferritin levels statistically correlated, but serum lymphocyte levels negatively correlated with the CT scores of lung involvement. We determined in our study that being older than 64 years of age and having a CT score of more than 11 at admission were the independent variables to predict the need for intensive care. Furthermore, the cut-off values to predict the need for intensive care were 20.7 for CRP, 805 for d-dimer, and 211 for ferritin.

Our study shows that laboratory and thoracic CT findings at the time of admission enable us to predict a severe disease course and the need for intensive care. We think that large-scale multi-center studies are needed.

## Figures and Tables

**Table 1.** Demographic and Disease Characteristics of Patients with Lung Involvement in Thoracic CT Examination (n = 143)

Characteristics	Total	CT Pattern			χ^2^/K–W	*P*
Ground-Glass Opacity	Crazy-Paving	Consolidation
**Age**						
Mean ± SD	54.24 ± 16.64	52.50 ± 16.03	62.47 ± 16.38	65.00 ± 19.48	7.150^a^	.028 *
Median	55 (19-91)	53 (19-89)	61 (37-88)	67 (31-91)		
**Gender**					0.926^b^	.629
Female n (%)	77 (53.8)	66 (55.0)	8 (53.3)	3 (37.5)		
Male n (%)	66 (46.2)	54 (45.0)	7 (46.7)	5 (62.5)		
**CT severity score**						
Right lung, superior lobe						
Mean ± SD	1.32 ± 1.05	1.22 ± 0.97	2.20 ± 1.26	1.25 ± 1.16	8.288^a^	.016*
Median	1 (0-5)	1 (0-3)	2 (0-5)	1.5 (0-3)		
Right lung, middle lobe						
Mean ± SD	1.13 ± 1.06	1.04 ± 0.99	1.73 ± 1.33	1.25 ± 1.16	4.088^a^	.129
Median	1 (0-4)	1 (0-4)	2 (0-4)	1.5 (0-3)		
Right lung, inferior lobe						
Mean ± SD	1.78 ± 1.15	1.77 ± 1.09	1.73 ± 1.33	2.00 ± 1.60	0.159^a^	.923
Median	2 (0-5)	2 (0-4)	2 (0-4)	2 (0-5)		
Left lung, superior lobe						
Mean ± SD	1.36 ± 1.02	1.29 ± 0.96	1.87 ± 1.30	1.50 ± 1.07	3.441^a^	.179
Median	1 (0-4)	1 (0-3)	2 (0-4)	2 (0-3)		
Left lung, inferior lobe						
Mean ± SD	1.85 ± 1.19	1.73 ± 1.09	2.27 ± 1.44	2.88 ± 1.55	6.972^a^	.031*
Median	2 (0-5)	2 (0-5)	2 (0-5)	3 (0-5)		
Total score						
Mean ± SD	7.40 ± 4.38	7.00 ± 4.08	9.80 ± 5.25	8.88 ± 5.69	3.960^a^	.138
Median	7 (1-21)	7 (1-16)	8 (3-21)	9 (2-18)		
**Biochemichal parameters**						
CRP						
Mean ± SD	34.10 ± 43.83	27.12 ± 36.44	58.44 ± 32.72	93.28 ± 90.29	20.836^a^	<.001*
Median	16.7 (0.2-233)	13.25 (0.2-186.5)	53 (13.9-122.4)	69.8 (7.7-233)		
D-dimer						
Mean ± SD	778.87 ± 1083.81	679.6 ± 782.3	917.0 ± 681.2	2009.5 ± 3248.8	13.345^a^	.001*
Median	512 (45-10.000)	453 (45-5990)	842 (356-3208)	862.5 (242-10.000)		
Ferritin						
Mean ± SD	283.68 ± 342.26	256.71 ± 321.19	457.39 ± 485.68	362.51 ± 257.99	6.133^a^	.047*
Median	176 (6-2000)	152.85 (6-2000)	399 (40-1815)	264 (72.1-778)		
Lymphocyte						
Mean ± SD	1.58 ± 0.64	1.60 ± 0.65	1.50 ± 0.69	1.51 ± 0.57	0.492^a^	.782
Median	1.51 (0.41-4.14)	1.55 (0.41-4.14)	1.34 (0.51-3.09)	1.45 (0.67-2.41)		
**Need for intensive care**					7.527^b^	.023*
Yes n (%)	19 (13.3)	12 (10.0)	4 (26.7)	3 (37.5)		
No n (%)	124 (86.7)	108 (90.0)	11 (73.3)	5 (62.5)		
**Final situation**					12.183^b^	.002*
Exitus n (%)	10(7.0)	6(5.0)	1(6.7)	3(37.5)		
Discharge n (%)	133 (93.0)	114 (95.0)	14 (93.3)	5 (62.5)		

^*^ *P* < .05; statistically significant;^ a^K–W, Kruskal–Wallis test; ^b^χ^2^, chi-square (Pearson’s chi-square) test.

CT, computed tomography; CRP, C-reactive protein; SD, standard deviation.

**Table 2.** The Relationship Between Age and Biochemistry Values and BT Involvement Score

Biochemical Parameters		CT Severity Scores					
Right Lung, Superior Lobe	Right Lung, Middle Lobe	Right Lung, Inferior Lobe	Left Lung, Superior Lobe	Left Lung, Inferior Lobe	Total
**CRP**	*r*	0.550	0.535	0.469	0.563	0.555	0.670
*P*	<.001*	<.001*	<.001*	<.001*	<.001*	<.001*
**D-Dimer**	*r*	0.334	0.339	0.271	0.273	0.301	0.379
*P*	<.001*	<.001*	.001 *	.001 *	<.001*	<.001*
**Ferritin**	*r*	0.285	0.239	0.274	0.277	0.256	0.311
*P*	.001 *	.004 *	.001 *	.001 *	.002 *	<.001*
**Lymphocyte**	*r*	−0.119	−0.182	−0.174	−0.118	−0.259	−0.218
*P*	.158	.029 *	.038 *	.159	.002 *	.009 *
**Age**	*r*	0.340	0.416	0.249	0.214	0.284	0.372
*P*	<.001*	<.001*	.003 *	.010 *	.001 *	<.001*

^*^ *P* < .05 statistically significant; *r*, Spearman correlation test.

CT, computed tomography; CRP, C-reactive protein.

**Table 3.** Independent Factors Associated with Intensive Care Need and Mortality (Multivariate Logistic Regression Analysis Results)

Factors	Intensive Care Need			Factors	Mortality		
Category	OR (95% CI)	*P*	Category	OR (95% CI)	*P*
Age	<65	Reference (1)		Age	<65	Reference (1)	
≥65	5.80 (1.37-24.51)	.017 *	≥65	7.88 (1.17-52.97)	.034 *
CT pattern	Ground-glass opacity	Reference (1)		CT pattern	Ground-glass opacity	Reference (1)	
Crazy-paving and consolidation	4.29 (0.82-22.44)	.085	Crazy-paving and consolidation	1.78 (0.27-11.67)	.548
CT score	<11	Reference (1)		CT score	- **	1.36 (1.07-1.73)	.012 *
≥11	20.60 (3.79-111.92)	<.001*
CRP	<21	Reference (1)		CRP	<21	Reference (1)	
≥21	1.16 (0.20-6.72)	.865	≥21	3.14 (0.28-35.34)	.355
d-dimer	≤805	Reference (1)		d-dimer	≤805	Reference (1)	
>805	1.05 (0.23-4.89)	.949	>805	3.78 (0.47-30.14)	.209
Ferritin	≤211	Reference (1)		Ferritin	≤211	Reference (1)	
>211	3.97 (0.87-18.23)	.076	>211	2.13 (0.31-14.53)	.440

^*^ *P* < .05 statistically significant; logistic regression (method = enter); odds ratio (OR) are presented with their 95% CI and the *P*-value; ^**^Since there was no mortality in the CT involvement score group of less than 11 in the CT involvement score classification, the CT categorical classification, and total CT score were included in the modeling.

CT, computed tomography; CRP, C-reactive protein.
